# Morphology and mechanical behavior of diatoms in wet and dry states studied using nano-XCT

**DOI:** 10.1186/s12915-025-02341-5

**Published:** 2025-08-05

**Authors:** Qiong Li, Jürgen Gluch, Zhongquan Liao, André Clausner, Przemysław Dąbek, Ehrenfried Zschech

**Affiliations:** 1 Fraunhofer Institute for Ceramic Technologies and Systems IKTS, Maria-Reiche-Str. 2, 01109 Dresden, Germany; 2 Institute of Physics, Brandenburg University of Technology Cottbus-Senftenberg, Faculty 1Konrad-Zuse-Str. 1, 03044 Cottbus, Germany; 3 Institute of Marine and Environmental Sciences, University of Szczecin, Mickiewicza 16, 70-383 Szczecin, Poland; 4 Leibniz Institute of Polymer Research Dresden, Hohe Str. 6, 01069 Dresden, Germany

**Keywords:** Diatom, 3D visualization, X-ray tomography, In situ micromechanical behavior

## Abstract

**Background:**

Diatoms are widely studied biological objects because of their large variety of geometric shapes and their unique physical and chemical properties. They survive widely in nature within moisture. Imaging the diatoms three dimensionally in moisture and correlating their mechanical behavior is an interesting and challenging topic.

**Results:**

Here, the morphology and mechanical properties of diatoms were studied in wet state and then in dry state. A customized sample holder was integrated into a laboratory transmission X-ray microscope to image the morphology changes and volume shrinkage of the diatom while transitioning from the wet to the dry state. The measured volume shrinkage of a single diatom cell of *Actinocyclus* sp. is about 0.16. By performing an in-situ micromechanical experiment in both states, the maximal loading force of a single *Actinocyclus* sp. was determined until cracking appeared and compared in both states. This value is in the range of several hundred µN in the wet state and single-digit mN in the dry state. The normalized stiffness of the studied diatoms is significantly higher in the dry state than in the wet state. 2D radiograph and 3D tomography imaging of the diatoms reveal the different locations for crack propagation in both states.

**Conclusions:**

Our study supplies the important imaging method, the structure and functional information of the diatoms for future studies on diatoms in moisture but also in dry state. This information can help design bio-inspired materials and even in the development of bio-sustainable materials.

**Supplementary Information:**

The online version contains supplementary material available at 10.1186/s12915-025-02341-5.

## Background

Diatoms are single eukaryotic organisms that can be found almost everywhere in nature as long as there is moisture and light available. Each diatom species lives in a uniquely shaped “house of glass,” which is made of silica. It is called either frustule or shell. On the one hand, due to its outstanding mechanical robustness, the diatom frustule protects the diatom cell from predators or from enzymatic disintegration [1, 2], while on the other hand, due to its porous microstructure, it allows an efficient interaction with the environment. Furthermore, diatom frustules exhibit an extraordinary diversity of shapes and hierarchical designs on micro- and nano-scales [3, 4]. The variety of geometrical shapes and the tailored structure of diatom frustules demonstrate the brilliance of the evolution-based hierarchical design of natural objects at the micro- and nanoscale. Additionally, the study of the morphology of diatoms provides fundamental knowledge for diatom biology, silica biomineralization, bio-inspired material design, biocompatibility materials, bio-sustainable materials, and biofuel generation and so on [5–15].

The morphology of diatoms has been widely studied using a broad range of characterization techniques including scanning electron microscopy (SEM), transmission electron microscopy (TEM) [11, 16–18], and confocal light microscopy (LM) [19, 20]. SEM and TEM provide information about the ultrastructure of surfaces and the inner structure of diatoms. For electron microscopy studies of the inner structure of diatoms, the preparation of cross-sections is required, i.e., the siliceous skeleton is exposed to mechanical, thermal, and/or chemical treatments that destroy the diatoms. To study the cells in their native state, without interference caused by chemical fixatives or contrasting agents, advanced cryo lift-out microscopy techniques and workflows have been developed and applied, particularly for cryo-LM, cryo focused ion beam (FIB)/SEM, and cryo-TEM [11, 20–24]. The study of the 3D morphology of the whole diatoms in wet state is rare. Hamm et al. [1] used a glass needle to compress the wet diatoms to get the mechanical properties of the diatoms two dimensionally. However, the morphology of the diatom changing with the compression has not been illustrated thoroughly. Savoia et al. [23] presented an approach for obtaining the 3D shape of the diatoms by digital holographic microscopy. They estimated the 3D shape of diatom cells, however, without detailed morphology information. Haan et al. [24] revealed the 3D surface morphology and simultaneously with mechanical property (stiffness) by using AFM to study the diatoms in wet state. Due to the low indentation depth, their study was on the surface of the silica wall (frustules) with mapping length about 40 µm, which is part of the whole diatoms (*Stephanopyxis turris*). Since the length of the whole *Stephanopyxis turris* is more than 100 µm, their study is not for the whole diatom.

Laboratory transmission X-ray microscopy and nano X-ray computed tomography (XCT) provide the unique opportunity to investigate the internal and external structure of the whole diatom frustules with high resolution non-destructively [25, 26], i.e., without destructive sample preparation like cutting, chemical fixation, or staining of contrasting agents for the diatom frustules. Hamm et al. [1] determined the mechanical loading response of centric diatom cells and pennate diatom cells in the wet state using glass microneedles to load and to break them. The maximum loading force was in the range from about 90 µN to about 700 µN. Recently, an in situ micromechanical experiment with uniaxial loading (compression test) combined with 3D imaging using nano-XCT was carried out to investigate the mechanical behavior of the whole frustules in the dry state [18]. For frustules from genera of *Ellerbeckia* and *Melosira*, the maximal loading force, at which the frustule structure mechanically collapses in the dry state, was determined to be in the mN range.

The aim of this paper is to study the 3D morphology of diatom cells under more realistic habitat-like conditions, i.e., in wet state, and to compare the results with respective ones in the dry state by customized sample holder integrated into nano-XCT system. Simultaneously, the changes of the mechanical properties while transforming from the wet state into the dry state are studied in situ. Particularly, whole diatom frustules and their intracellular structures are imaged at laboratory atmosphere (no vacuum) in dry and wet states three dimensionally. Besides, based on the previous studies, it was found that the force to break the whole single frustule in wet state is in the range of hundreds of µN [1], while the respective force is much higher in the dry state [18]. Therefore, the current study in addition focuses on the verification and the root causes of these findings. Furthermore, the mechanical behavior is compared both in 2D and 3D to reveal the cracks and their propagation. This information can help design bio-inspired materials and even in the development of bio-sustainable materials.

## Results

### 3D morphology and volume analysis

**Fig. 1:**
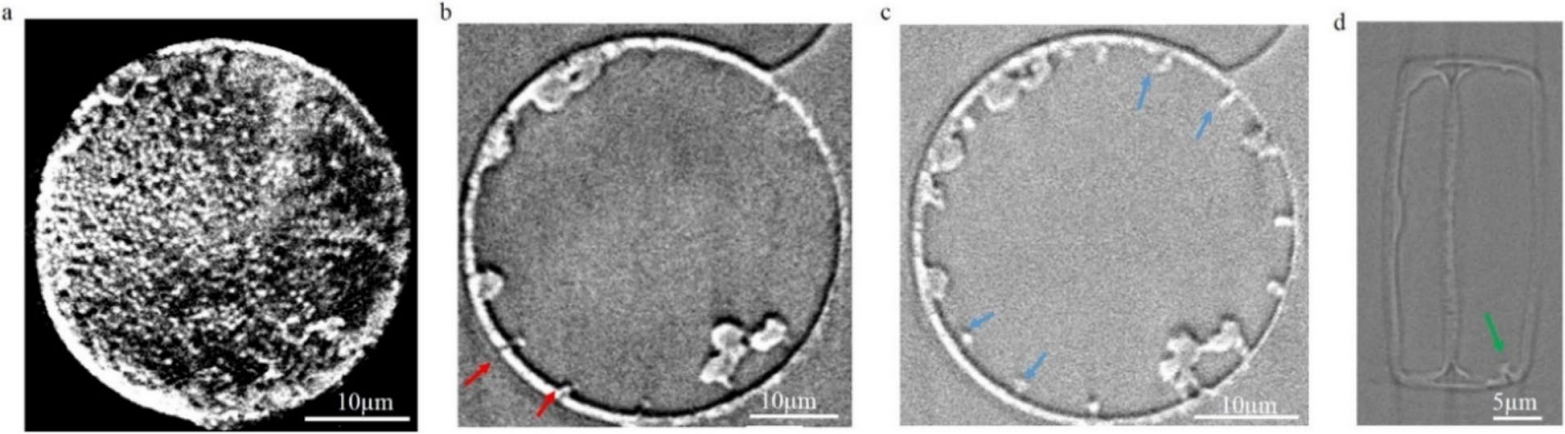
Virtual cross-sections of the *Actinocyclus* sp. in dry state, based on 3D nano-XCT data. **a** Valve surface of *Actinocyclus* sp. with radiate areolae. **b**, **c** Valve view of rimoportulae (labiate process) presenting at margin of the valve face with the simple, round aperture (**b**) and the shape of the lips on the internal valve face (**c**); this type of diatom has about 14–16 rimoportulae. **d** Girdle view of *Actinocyclus* sp. Red arrows: the opening of labiate processes, blue arrows: the rimoportulae (valve view of internal part of the labiate processes), green arrow: pseudonodulus

**Fig. 2:**
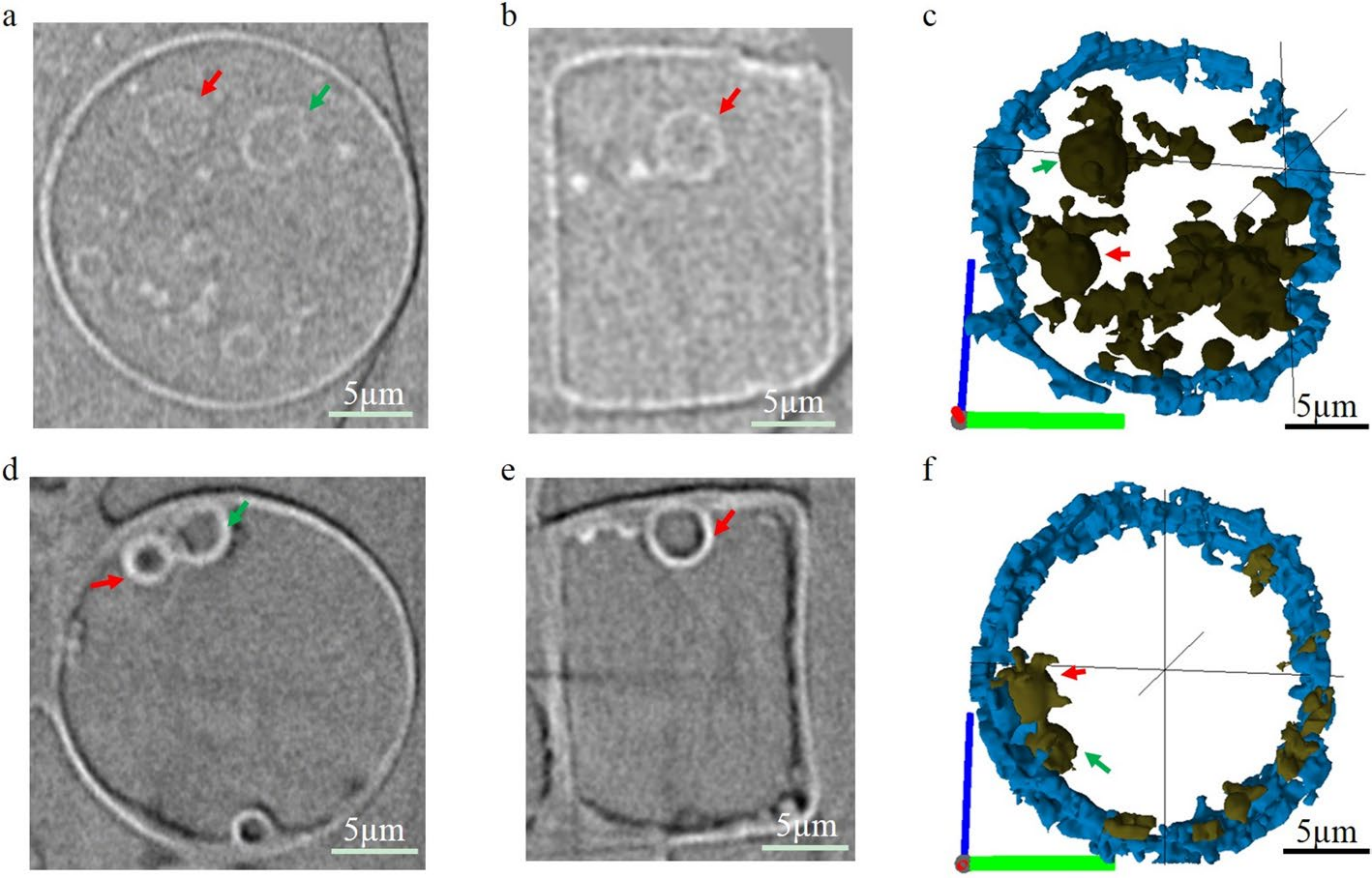
Virtual cross-sections of the same *Actinocyclus* sp. in wet and dry state based on 3D nano-XCT data. **a**, **b** Valve view (**a**) and girdle view (**b**) of *Actinocyclus* sp. in wet state with intracellular structures distribution in a diatom cell. **c** 3D rendering of the intracellular structures (grey-yellow) and part of the frustule wall (blue) from the *Actinocyclus* sp. cell in the wet state. **d**, **e** Valve view (**d**) and (**e**) girdle view of the same *Actinocyclus* sp. cell in the dry state with the intracellular structures sticking to the frustule wall. **f** 3D rendering of the intracellular structures (grey-yellow) and part of the frustule wall (blue) from the *Actinocyclus* sp. cell in dry state. Red and green arrows: the intracellular structures

Virtual cross-sections through the *Actinocyclus* sp. with a valve diameter of about 40 µm in dry state extracted from 3D nano-XCT images are shown in Fig. 1. The valve faces have radiate and coarse areolae (black shadow and white spots) (Fig. 1a). There are 14 to 16 rimoportulae for this type of diatom, located equally spaced along the valve margins (arrows in Fig. 1b, c, Additional file 1: Video S1). On the external valve face, the rimoportula opening is a simple, round aperture at the valve surface (Fig. 1b) and forms a tube to connect the inner cell with the outside environment. On the internal valve face, the rimoportula openings have the shape of lips (Fig. 1c). Figure 1d shows a girdle view of *Actinocyclus* sp. The pseudonodulus is visible with the position on the valve/mantle interface (green arrow in Fig. 1d). Figure 2 compares the wet state and dry state of *Actinocyclus* sp. with a valve diameter of about 20 µm. In the wet state, the intracellular structures are well distributed inside the diatom cell (arrows in Fig. 2a, b, grey-yellow part in Fig. 2c, Additional file 2: Fig. S1 and Additional file 3: Video S2), while in the dry state, these structures stick to the frustule wall; some of them are even broken and without any morphology (arrows in Fig. 2d, e, grey-yellow part in Fig. 2f, Additional file 2: Fig. S1 and Additional file 4: Video S3). Since the limited resolution of the nano-XCT system (about 120 nm with 65 µm field of view), much of the detail of the silica wall (e.g., radiate and coarse areolae or nano-scale pore structures) and intracellular structures were not illustrated for the *Actinocyclus* sp. with a valve diameter of about 20 µm. Based on 3D nano-XCT data and the calculation using Eq. 1, a volume shrinkage rate of 0.16 ± 0.05 was determined for the *Actinocyclus* sp. diatoms after evaporation of the water (Table 1).

**Table 1:** Parameters of *Actinocyclus* sp. and volume comparison from dry to wet states

Diatoms	Dry state			Wet state			Shrinkage
	*R* (µm)	*L* (µm)	*V*_t_ (µm^3^)	*R* (µm)	*L* (µm)	*V*_t_ (µm^3^)	*S*
**01**	24.6 ± 0.3	19.6 ± 0.4	8789.4	25.4 ± 0.5	26.4 ± 0.3	11,394.7	0.23
**02**	22.4 ± 0.3	16.1 ± 0.4	6398.4	22.8 ± 0.2	16.9 ± 0.3	7057.1	0.10
**03**	23.5 ± 0.5	11.6 ± 0.4	5009.6	24.0 ± 0.3	12.0 ± 0.5	5946.0	0.16
**04**	22.0 ± 0.4	18.4 ± 0.4	6989.0	22.9 ± 0.3	20.3 ± 0.8	7922.7	0.12
**05**	24.0 ± 0.3	15.7 ± 0.5	7186.6	24.4 ± 0.4	20.1 ± 0.3	9035.1	0.20

*R valve diameter of the *Actinocyclus* sp*.*, *L* length of the *Actinocyclus* sp. between two valve faces, *V*_*t*_ volume derived from the tomography data

### In situ mechanical study of ***Actinocyclus*** sp. in wet and dry states

**Fig. 3:**
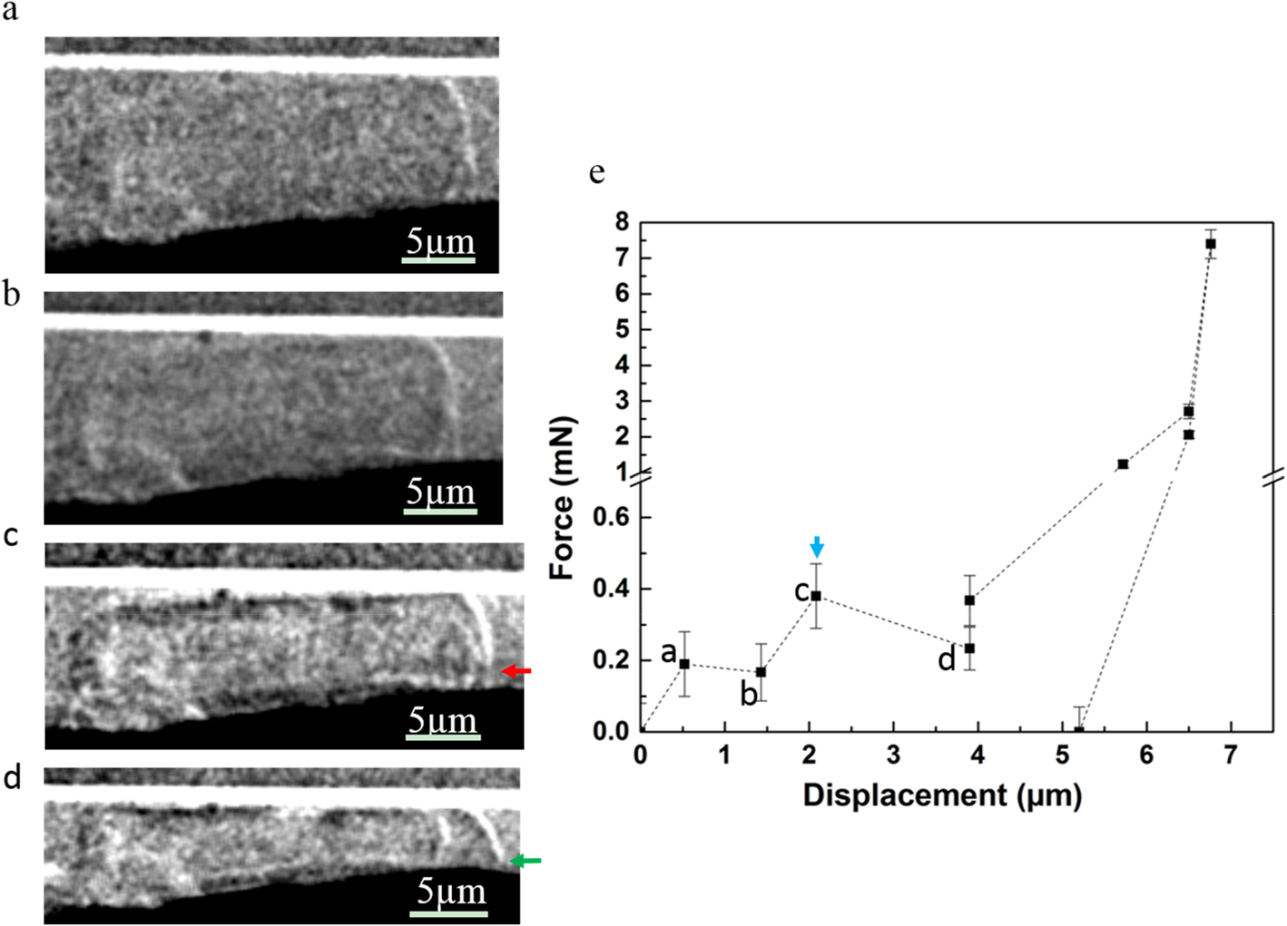
Typical in-situ compression test on *Actinocyclus *sp. in wet state.** a** to **d** Radiographs recorded during the micromechanical test. **e** Load–displacement curve. The indicated data points (**a**, **b**, **c**, **d**) in **e** correspond to the radiographs **a** to **d**. Red arrow: the horizontal crack initiates from the connection regions between valve face and the girdle band and propagates perpendicular to the compression direction with a size about 1 µm wide and 7 µm long. Green arrow: the diatom cell collapsed and delaminated. Blue arrow: the maximal force (*F*_max_) before the first crack

Diatoms of *Actinocyclus* sp. with a valve diameter of about 22 µm were mechanically tested in wet state and in dry state by applying the in-situ compression test using a diamond flat punch with a diameter of about 120 µm. Figure 3a–d show 2D radiographs recorded during the mechanical test of the diatoms in wet state, from the first flat punch contact with the cell (Fig. 3a) until cracking appeared (Fig. 3c). The radiographs show the deformed diatom cell (Fig. 3d), and the cell at a maximal loading force of about 400 µN when the first cracking appeared (Fig. 3c). The red arrow in Fig. 3c shows the crack with a width of about 1 µm and a length of about 7 µm. The horizontal crack initiates from the connection region between the valve face and girdle band, then propagates perpendicular to the compression direction in wet state. Further compression (Fig. 3d) makes the whole diatom cell collapse and delaminate (green arrow in Fig. 3d). The indicated data points (a, b, c, d) in Fig. 3e correspond to the radiographs shown in Fig. 3a to d. The mechanical tests on the diatom cells in wet state (Table 2) result in a maximal loading force of one single frustule of about 390 µN, as an average value from 4 diatoms’ measurements. This value is consistent with the results published in the paper of Hamm et al. [1], in which the force required to break the living diatom frustule by a glass needle is in the range from tens to hundreds of µN.

**Table 2:** The maximal loading force (F_max_) and the compound cell modulus $M_c$ of the *Actinocyclus* sp. in wet state and in dry state

	Sample	Parameters						
		Valve Diameter	*F*_max_	*F*_e_	A	⊿_l_	H	M_c_
		µm	mN	mN	µm^2^	µm	µm	mN/µm^2^
Wet state	01	23.1 ± 0.2	0.40 ± 0.08	0.36 ± 0.08	409 ± 9	0.94	23.1 ± 0.2	0.022
	02	21.2 ± 0.2	0.35 ± 0.04	0.32 ± 0.04	426 ± 37	0.83	21.8 ± 0.2	0.020
	03	22.5 ± 0.5	0.40 ± 0.08	0.36 ± 0.08	357 ± 11	0.51	9.5 ± 0.7	0.019
	04	21.3 ± 0.3	0.40 ± 0.07	0.36 ± 0.07	363 ± 18	1.07	20.2 ± 0.2	0.018
Dry state	05	20.3 ± 0.1	1.86 ± 0.19	1.67 ± 0.19	443 ± 21	0.60	22.7 ± 0.2	0.137
	06	23.0 ± 0.1	1.87 ± 0.20	1.69 ± 0.20	378 ± 18	0.55	22.0 ± 0.9	0.180
	07	21.8 ± 0.1	1.83 ± 0.19	1.65 ± 0.19	315 ± 19	0.48	21.6 ± 0.5	0.229
	08	21.5 ± 0.1	1.87 ± 0.40	1.68 ± 0.4	419 ± 20	0.49	22.6 ± 0.5	0.187

**Fig. 4:**
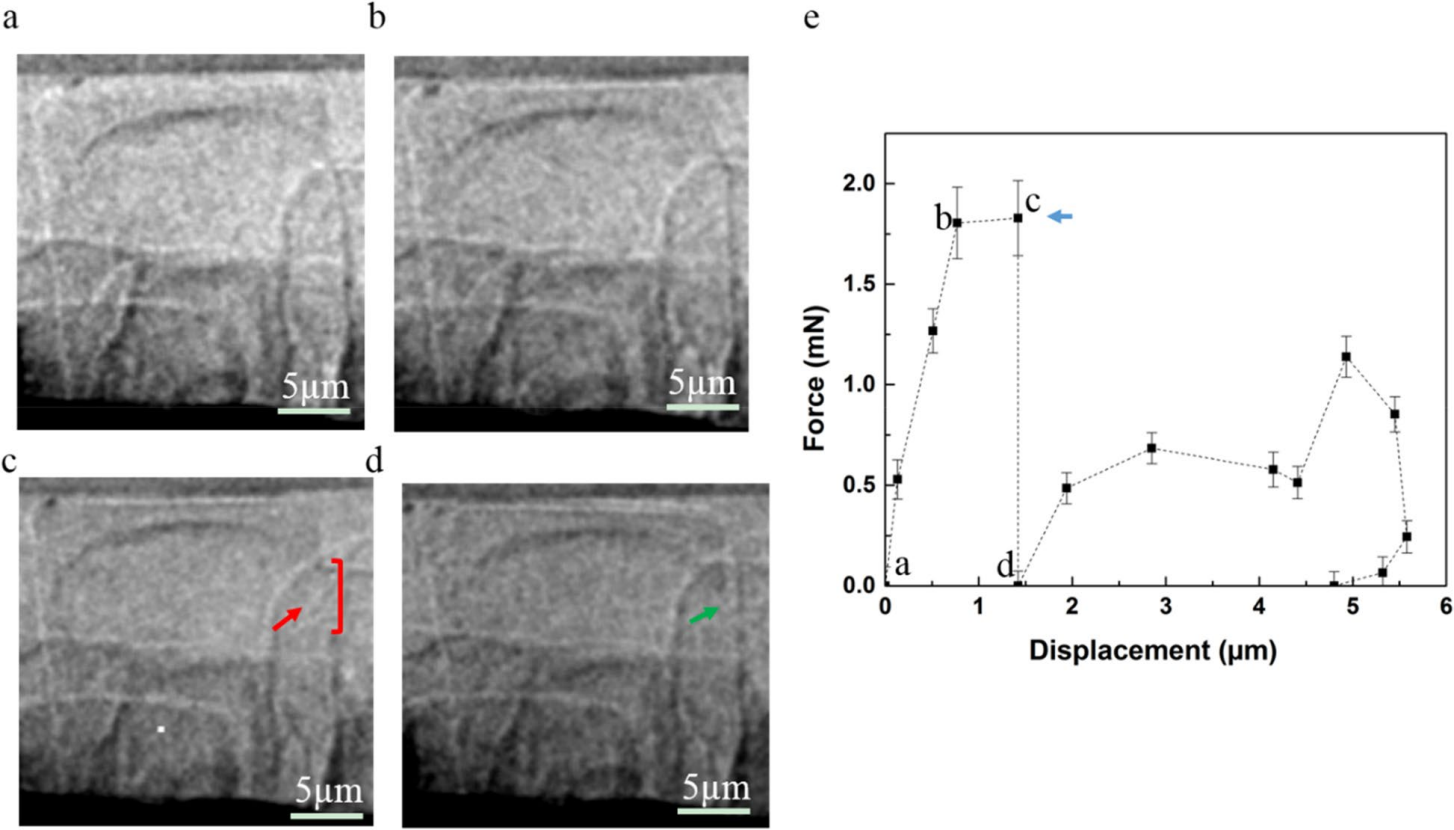
Typical in-situ compression test on *Actinocyclus *sp. in dry state.** a** to **d** Radiographs recorded during the micromechanical test. **e** Load–displacement curve. The indicated data points (**a**, **b**, **c**, **d**) in **e** correspond to the radiographs **a** to **d**. Red arrows: the micro-crack within the compression test. Red right bracket: the crack opening and size. Green arrow: the upper valve face collapsed until it is supported again by the girdle band. Blue arrow: the maximal force (*F*_max_) before the first crack

Figure 4a–d show 2D radiographs recorded during the mechanical test of the diatoms in dry state from the first flat punch contact with the cell (Fig. 4a) until cracking appeared (Fig. 4c). The position where the crack appeared is indicated with a red arrow in Fig. 4c; there is an uncontinuous area on the diatom frustule wall with about a 5 µm crack (right bracket) showing the crack area compared with the corresponded continuous area in the diatom frustule wall in Fig. 4a, b. Further compression causes the upper valve face to collapse until it is supported again by the girdle band (green arrow in Fig. 4d), and at the same time, the force drops (data point d in Fig. 4e). The indicated sketch in additional file 2: Fig. S7 shows clearly the crack behavior during the compression test. The indicated data points (a, b, c, d) in Fig. 4e correspond to the radiographs shown in Fig. 4a to d. The repeated mechanical tests on the diatom cells in dry state (Table 2) result in a maximal loading force of one single frustule of about 1.86 mN, as an average value from 4 diatoms’ measurements. This value is significantly higher than the respective value for diatoms in the wet state.

**Fig. 5:**
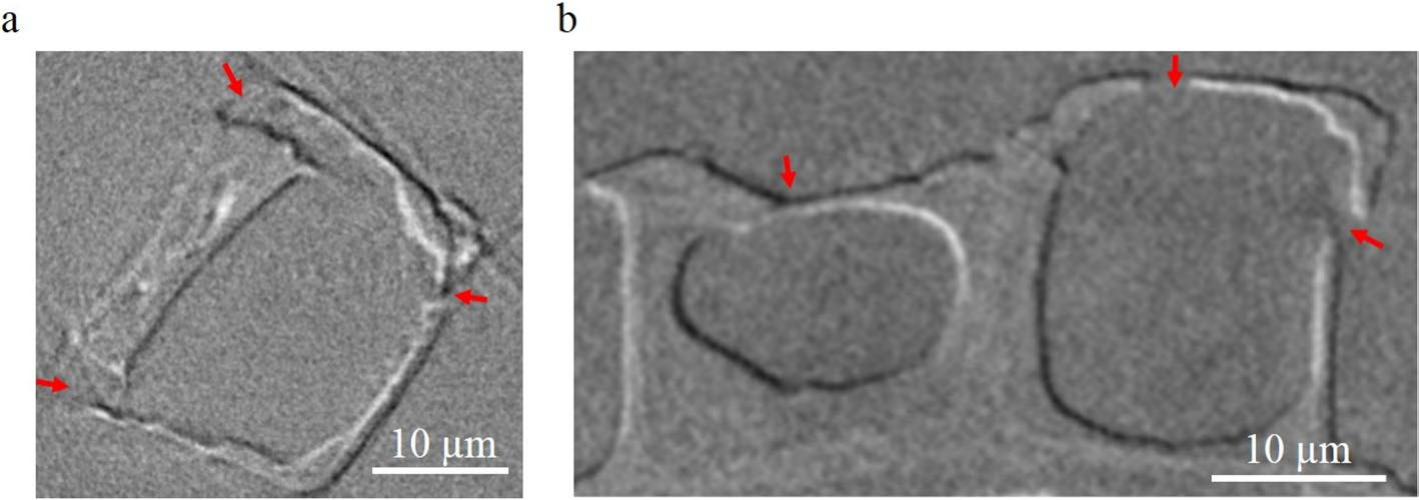
The images of *Actinocyclus* sp. after the in-situ compression test.** a**, **b** Virtual cross-section of the *Actinocyclus *sp. after the micromechanical test in the wet state (**a**) and in the dry state (**b**). Red arrows: positions of the microcracks

The images of the *Actinocyclus* sp. after the in situ compression test show the positions where the micro-cracks propagate (red arrows in Fig. 5, Additional file 2: Fig. S8). In the wet state (Fig. 5a, Additional file 2: Fig. S8, Additional file 5: Video S4 and Additional file 7: Video S6), the compression causes a detachment in the connection region between valve face and the girdle band, i.e., these are the weak regions of the diatom in the wet state. Obviously, in Additional file 2: Fig. S8a and b, there is a crack (red dashed lines) with a length about 20 µm, which crosses through the valve face and girdle band. In the dry state (Fig. 5b), both valve face and girdle band are broken under compression, i.e., these are the weak regions. The observation is similar for the fossil frustules without any organic materials inside (Additional file 2: Fig. S2). Additionally, the nano-XCT imaging (Additional file 2: Fig. S8c and d, Additional file 6: Video S5 and Additional file 8: Video S7) after the compression test shows the crack propagations three dimensionally. One crack crosses through the valve face, and another crack crosses through the girdle band. The results from the two states of the diatom cells indicate that the hydration state of the cells is a key factor determining the maximal loading force. In the wet state the maximal loading force needed is in the range of hundreds of µN (0.39 ± 0.02 mN), and in the dry state it is in the range of single-digital mN (1.86 ± 0.02 mN). For comparison, a compound cell modulus (${M}_{c}$) was introduced, i.e., a normalized stiffness value that considers the geometry of the diatom cell. This ${M}_{c}$ value is calculated according to Eq. 2. For the diatom cell studied, the compound cell modulus is 0.019 ± 0.001 mN/µm^2^ in the wet state and 0.183 ± 0.033 mN/µm^2^ in the dry state. That means the normalized stiffness of the diatom frustule is much higher in the dry state than in the wet state.

## Discussion

The morphology of *Actinocyclus* sp. diatom frustules was studied in wet and dry states using nano-XCT, without invasive sample preparation. A customized sample holder was designed, built, and integrated into an X-ray microscope to image the 3D morphology of the diatoms and to perform in-situ micromechanical experiments of the diatoms. In the wet state, i.e., in near-natural conditions of the diatoms, the intracellular structures are distributed well inside the diatom cell containing cytosol. The diatom cells do not contain cytosol in the dry state, and most of the intracellular structures are broken or sticking on the diatom cell wall. The volume shrinkage caused by the evaporation of the water while changing from the wet state to the dry state is 0.16 ± 0.05.

In situ mechanical compression tests at *Actinocyclus* sp. frustules performed in the nano-XCT tool reveal maximum loading forces of hundreds of µN in the wet state and in the mN range in the dry state. For *Actinocyclus* sp*.* frustules with a diameter of about 22 µm*,* the *F*_max_ is about 0.39 mN for the wet state and about 1.86 mN in the dry state. For the specific diatoms with valve diameter size of about 20 µm, there is almost a 5 times difference for the maximum loading forces compared from the wet state to dry state. The calculation of the compound cell modulus ${M}_{c}$ shows that the diatom cells are much stiffer in the dry state than in the wet state. There is almost 10 times the difference for the compound cell modulus Mc. That is 0.019 ± 0.001 mN/µm^2^ in the wet state compared to 0.183 ± 0.033mN/µm^2^ in the dry state. Furthermore, the weakest regions for fracture are on the valve faces or on the girdle band for the diatom cells in dry state, while the weakest region is the region between valve face and girdle band, the joint area for the diatom cells in the wet state. For the locations of crack propagation, the diatoms in dry state are similar to the *Melosira* frustule [18]. That is, crack initiates from one of the weakest regions and propagates through the valve face or girdle band. However, for the crack propagation in the wet state, it has totally different mechanism, that is crack initiates from one position of the connection regions of the valve faces and girdle bands regions, then propagates through the whole connection regions, that is, the valve face and girdle band are detached from each other after the compression test on the whole diatom cell.

The significant differences of the micromechanical behavior of diatom frustules in the two states discussed above can be explained with the hydrated state of the diatom cells and the organic material inside the diatom cell. For the diatom cell in the wet state, the organic material remains in the original location with the cytosol inside the diatom cell. During the transition from the wet state to the dry state, the organic material is relocated from the original location to the cell wall of the diatoms, which strengthens the cell wall. This dried material sticking at the side walls is also assumed to be the main reason for the different micro-crack propagations and the change in the fracture behavior. Another reason might be that the soft tissue serves as the buffer at the joint interface of silica shell in the wet state, explaining the reduced stiffness of the diatom in the wet state as a combined effect of soft joint tissue and silica shell. In total, clearly visible using the *M*_*c*_ (Table 2.), the whole *Actinocyclus* sp. cell becomes more rigid in the dry state because of the changes of the hydrated state of the cells and their material.

## Conclusions

Our customized sample holder was successfully integrated into the nano-XCT system to image the diatoms in a wet state. The characterization of the 3D morphology and volume change of the same diatom from wet state to dry state clearly shows the intracellular structure change and volume shrinkage. A correlative study of their mechanical properties reveals the different behavior of the diatoms in both wet and dry states. A single diatom of *Actinocyclus* sp. in a wet state with a diameter of about 22 µm can sustain a compression with a maximal loading force of about 0.39 mN; a single diatom of *Actinocyclus* sp. in a dry state with a diameter of about 22 µm can sustain a compression with a maximal loading force of about 1.86 mN; the normalized stiffness of diatoms in the dry state is much higher than in the wet state; 2D radiograph and 3D tomography imaging reveal the different locations for crack propagation, in the connection regions between the valve face and girdle band in the wet state, and at the thin area on the valve face and girdle band in the dry state. Our research provides valuable information and methods for the application of the nano-XCT in biological objects. Besides, the insights gained from the mechanical study can support the design of bio-inspired materials and the development of bio-sustainable alternatives.

## Methods

### The culturing of diatom cells

The *Actinocyclus* sp*.* are diatom species from Szczecin Diatom Culture Collection (SZCZ). To keep the diatom species for living a long time, the *Actinocyclus* sp. was grown in liquid medium in 250 ml’s Erlenmeyer flasks. The liquid solution is f/2 medium (Additional file 2: Table S1 [27]) with specific salinity (35 PSU).

### Experimental set-up for imaging of diatoms in wet state

For the imaging of the 3D morphology of diatom cells and for the study of their mechanical behavior in the wet state, a customized set-up—sample imaging holders for wet samples as well as in situ holders combined with a flat punch (micro-indenter)—were designed, built, and integrated into a nano-XCT tool (Additional file 2: Fig. S3 and S4). This setup allows to keep the diatoms in the moisture state for more than 3 days (Additional file 2: Fig. S3c, d, e), which is sufficient to perform the in-situ nano-XCT experiments. Additional file 2: Fig. S3a illustrates the integration of the sample holder in the X-ray microscope. The laboratory X-ray microscope (nanoXCT-100, Xradia, Concord/CA, USA) with a rotating anode X-ray source with Cu target was operated at a photon energy of 8 keV (Cu-Kα radiation).

### Sample holder and imaging of diatoms by nano-XCT

A sample holder made of brass and a polyimide sleeve was designed for holding wet diatoms (Additional file 2: Fig. S4). The compartment to hold the diatoms on top of the brass base has a diameter of 3.0 mm and a 0.4 mm thick cylindrical wall on the upper part, embedding the polyimide sleeve into the cylindrical wall. The top of this compartment is open for access to the micro-indenter tip (flat punch). More details are provided in Additional file 2: Fig. S3. A photo of the sample holder is provided in Additional file 2: Fig. S3b. The upper side of the sample holder, fixed with a polyimide tube (inner diameter, 2.9 mm; length, ~ 1.5 cm, Goodfellow Cambridge, Huntingdon, UK) reserves the water (Additional file 2: Fig. S3c). For loading the diatom sample in the sample holder, firstly, a needle with a diameter of 0.75 mm is mounted inside the sample holder set-up. Then, water is filled in the miniaturized water reservoir of the sample holder. Using a light microscope (AxioCam ICc 3, Carl Zeiss, Oberkochen, Germany), the cultured diatoms are carefully picked up and transferred to the needle tip. The transfer time should be kept as short as possible. After checking that the sample has been successfully put on the sample tip under an auxiliary visible-light microscope (also called pre-alignment microscope (PAM)), a gold fiducial marker is carefully positioned on the sample. This marker is used for the alignment of the individual images acquired at several tilt angles needed for a 3D tomographic reconstruction. Finally, a small amount of modelling clay (Pelikan, Hannover, Germany) is used to cover the upper opening of the miniature water reservoir. This customized sample holder keeps the diatoms in a wet condition for radiography (2D imaging) or tomography (3D imaging) (Additional file 2: Fig. S3d, e). The complete tomography data set consists of 401 (compression experiments) or 801 (3D morphology study) images, which are collected for a range of tilt angles of 180° with an exposure time of 180 s for each image. These images were aligned using a custom plugin in ImageJ [28] and subsequently reconstructed using the commercial software package of Xradia Inc[29].. The data analysis was carried out by ImageJ, Tomviz [30]or Ilastik [31], depending on the data quality needed for data analysis.

### Volume shrinkage

The following geometry parameters were derived from a diatom, based on nano-XCT data: valve diameter (R), length between two valve faces (L), and the volume of the whole *Actinocyclus* sp. diatom cells. The volume shrinkage rate (S) of the diatoms while transferring from wet state to dry state is calculated as:

1 $$S\;=\;1\;-\;\frac{Vt\;(dry\;state)}{Vt(wet\;state)}$$

### In situ compression test on diatoms in wet state in the nano-XCT tool

In situ micromechanical compression tests of the diatoms in wet state are performed by a micro-mechanical test setup (Additional file 2: Fig. S5 and S6) integrated into the nano-XCT tool (nanoXCT-100, Xradia, Concord/CA, USA). For the in situ micromechanical compression testing in wet state, the diatoms were enclosed in a small water reservoir that can reserve the water for about 1.5 h (Additional file 2: Fig. S5). Water was added as necessary, for example, just before the compression test started. The procedures to load the living diatom in the in situ micromechanical test set-up (Additional file 2: Fig. S5) are the same as described in the method section of diatom preparing for nano-XCT imaging. As shown in Additional file 2: Fig. S6, the whole compression set-up allows to acquire radiographs for a range of tilt angles up to 90° for limited angle tomography. The compression test set-up consists of a piezo-mechanical actuator, a force gauge, and two anvils. For this study, a diamond flat punch was used as the upper anvil. For the mechanical testing, a single diatom cell is selected and compressed between two flat surfaces (bottom: steel sample holder, top: diamond flat punch) by stepwise increasing the load. The loading force is determined, and a radiograph with 60 s or 90 s exposure time is recorded at each step. The 3D tomography data of the diatom samples consist of 401 images collected during 180° sample rotation with an exposure time of 120 s for each image. For the diatom in dry state, the living diatom was added on the needle tip with the same procedures but without a water reservoir. Then, the living diatom was naturally dried in air for at least more than 1 week. The in situ mechanical compression test was performed subsequently.

### Compound cell modulus (M_c_)

The normalized stiffness is calculated from:

2 $$M_c=\frac{\displaystyle\frac{Fe}A}{\displaystyle\frac{{\mathit\triangle}_{\mathit l}}H}$$

${F}_{e}$ is a force value that is equal to 90% of *F*_max_ in the non-cracking regime. *A* is the footprint area which is the cross-section of the diatom in horizontal direction at the position where the force is applied to the diatom cell, ${\Delta }_{l}$ is the deformation of the diatom cell after the flat punch initially contacted, and *H* is the height before the flat punch contacts the diatom cell.

## Supplementary Information

Additional File 1: Video S1-[Video of 3D rendering of the *Actinocyclus* sp. with a valve diameter about 40 µm in dry state].

Additional File 2: Figures S1-S8, Table S1. FigS1-[3D imaging and intracellular morphology change of the *Actinocyclus* sp. from wet state to dry state]. FigS2-[Cross-sections of diatom frustules, in-situ compression test in the nano-XCT tool]. FigS3-[Set-up for imaging of diatom cells in wet state]. FigS4-[In-house design of the sample holder]. FigS5-[In-situ compression test on diatoms in wet state]. FigS6-[Set-up of the in-situ compression test of the diatom cell in wet state]. FigS7-[The indicated sketch of the studied diatom in Fig. 4 for the in-situ compression test on *Actinocyclus* sp. in dry state]. FigS8-[3D volume rendering images of the whole diatom frustule after in-situ compression test in the nano-XCT tool in both states]. TableS1-[Nutrient solution for diatoms: 1L recipe].

Additional File 3: Video S2-[Video of 3D rendering of the *Actinocyclus* sp. cell in the wet state].

Additional File 4: Video S3-[Video of 3D rendering of the *Actinocyclus* sp. cell in the dry state].

Additional File 5: Video S4-[Video of 3D rendering of the *Actinocyclus* sp. cell after in-situ compression test in wet state].

Additional File 6: Video S5-[Video of 3D rendering of the *Actinocyclus* sp. cell after in-situ compression test in dry state].

Additional File 7: Video S6-[Video of virtual cross-section of the *Actinocyclus* sp. cell after in-situ compression test in wet state].

Additional File 8: Video S7-[Video of virtual cross-section of the *Actinocyclus* sp. cell after in-situ compression test in dry state].

## Data Availability

All data generated or analyzed to support the conclusions of this article are included within the article and its additional files.
