# Supplementary material for: Morphology and mechanical behavior of diatoms in wet and dry states studied using nano-XCT
Source: BMC Biol. 2025 Aug 5;23:239. doi: 10.1186/s12915-025-02341-5 (PMC12326850; doi:10.1186/s12915-025-02341-5)
Supplement: Supplementary file 2 — Additional File 2: Figures S1-S8, Table S1. FigS1-[3D imaging and intracellular morphology change of the Actinocyclus sp. from wet state to dry state]. FigS2-[Cross-sections of diatom frustules, in-situ compression test in the nano-XCT tool]. FigS3-[Set-up for imaging of diatom cells in wet state]. FigS4-[In-house design of the sample holder]. FigS5-[In-situ compression test on diatoms in wet state]. FigS6-[Set-up of the in-situ compression test of the diatom cell in wet state]. FigS7-[The indicated sketch of the studied diatom in Fig. 4 for the in-situ compression test on Actinocyclus sp. in dry state]. FigS8-[3D volume rendering images of the whole diatom frustule after in-situ compression test in the nano-XCT tool in both states]. TableS1-[Nutrient solution for diatoms: 1L recipe]. [file 12915_2025_2341_MOESM2_ESM.docx]

**Additional file 2**

**Morphology and mechanical behavior of diatoms in wet and dry states studied using nano-XCT**


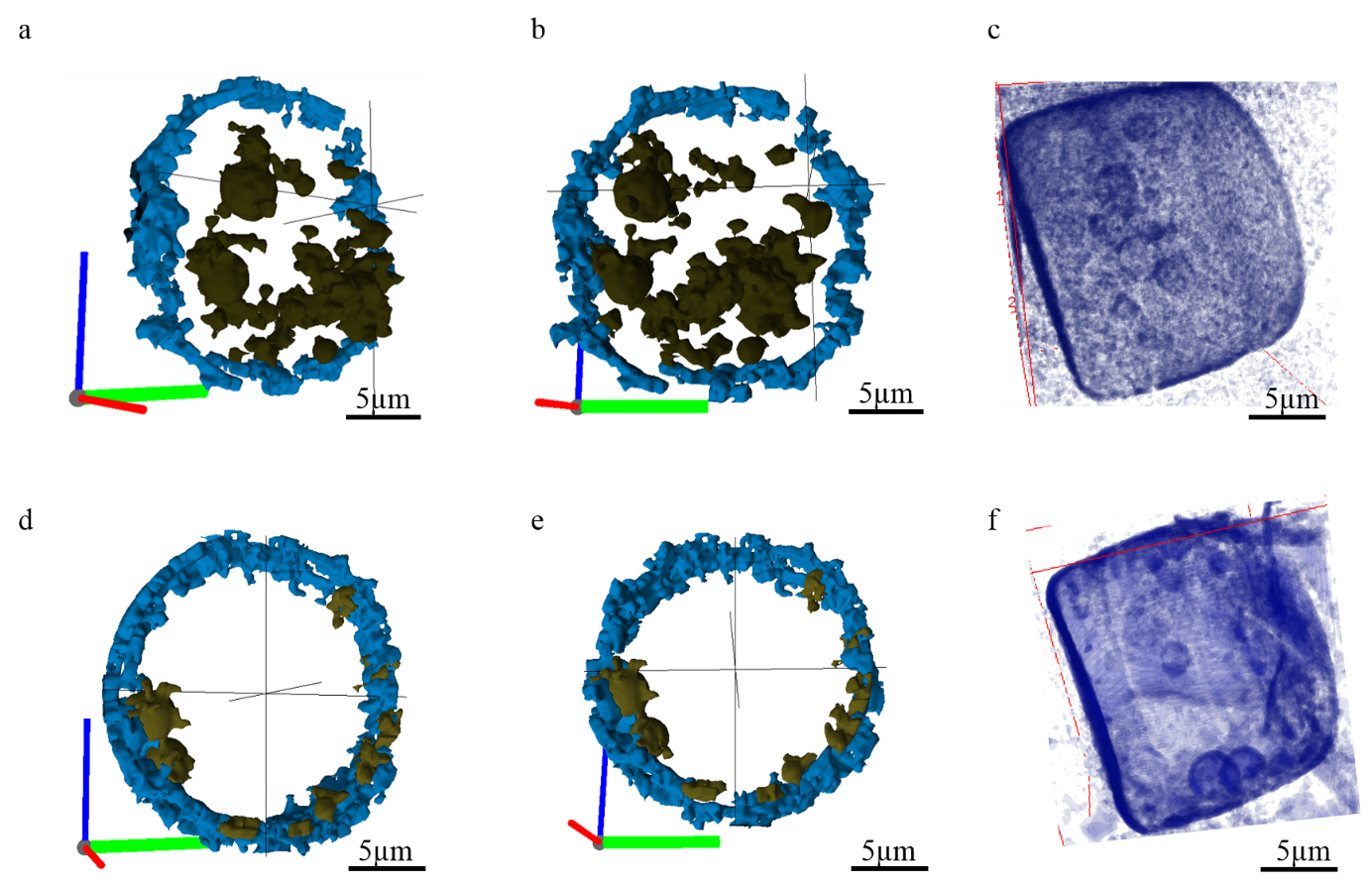


**Figure S1. 3D imaging and intracellular morphology change of the *Actinocyclus* sp. from wet state to dry state. a, b** Selected positions of the 3D segmented of the *Actinocyclus sp.* in wet state (intracellular structures in grey yellow; part of the frustules wall in blue). **d, e** Corresponding to the positions in (**a, b**) of the 3D segmented images of the same *Actinocyclus* sp*.* in dry state (intracellular structures in grey yellow; part of the frustules wall in blue). **c, f** are the 3D volume rendering images of the whole diatom frustule wall and intracellular structures in wet state (**c**) and dry state (**f**).


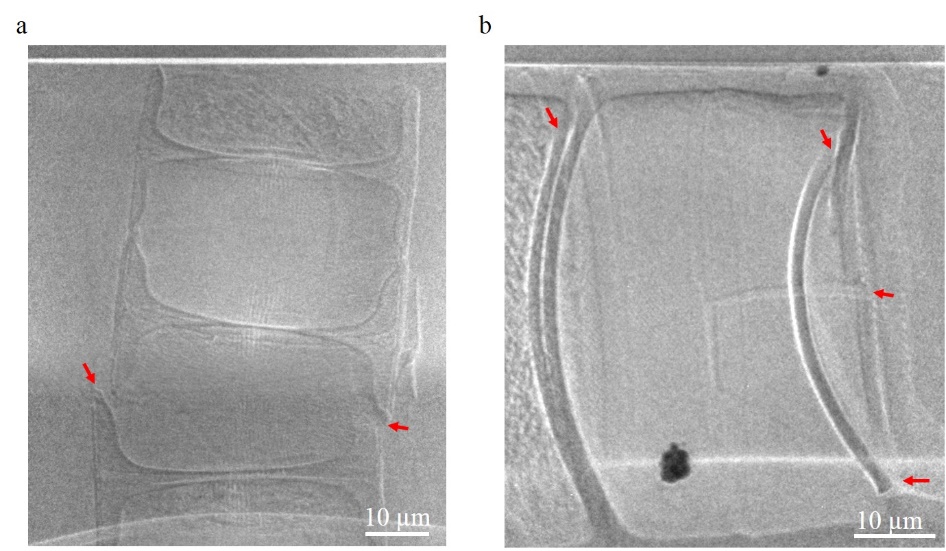


**Figure S2. Cross-sections of diatom frustules, in-situ compression test in the nano-XCT tool.** **a** The cracks of a *Melosira* frustule with a cylindrical diameter 46 µm during the compression test. **b** The cracks of a *Ellerbeckia* frustule with a cylindrical diameter of 62.5 µm during the compression test. Red arrows: the positions of the cracks taken place.


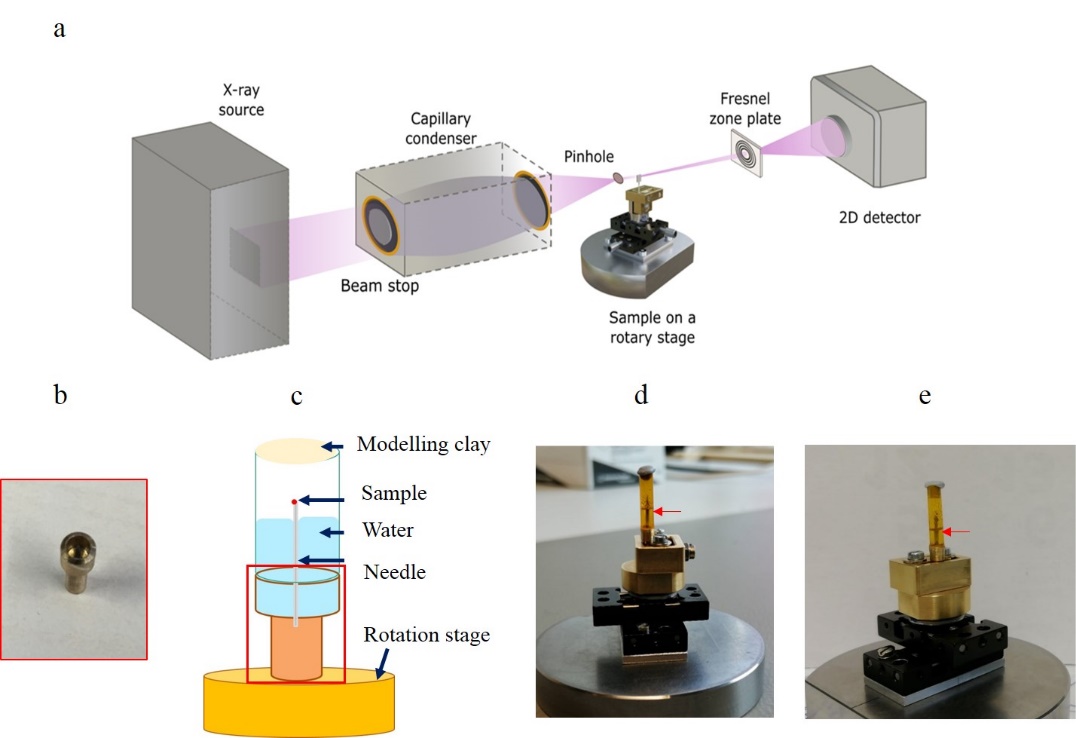


**Figure S3. Set-up for imaging of diatom cells in wet state**. **a** Scheme of the nano-XCT experiment in a laboratory transmission X-ray microscope setup operated at 8 keV (Cu-Kα radiation). **b** Photo of the sample holder part (red rectangle in c). **c** Scheme of the sample mounted on a rotary stage. **d, e** Water reservoir level (red arrow) in the set-up before (**d**) and after three days (**e**).


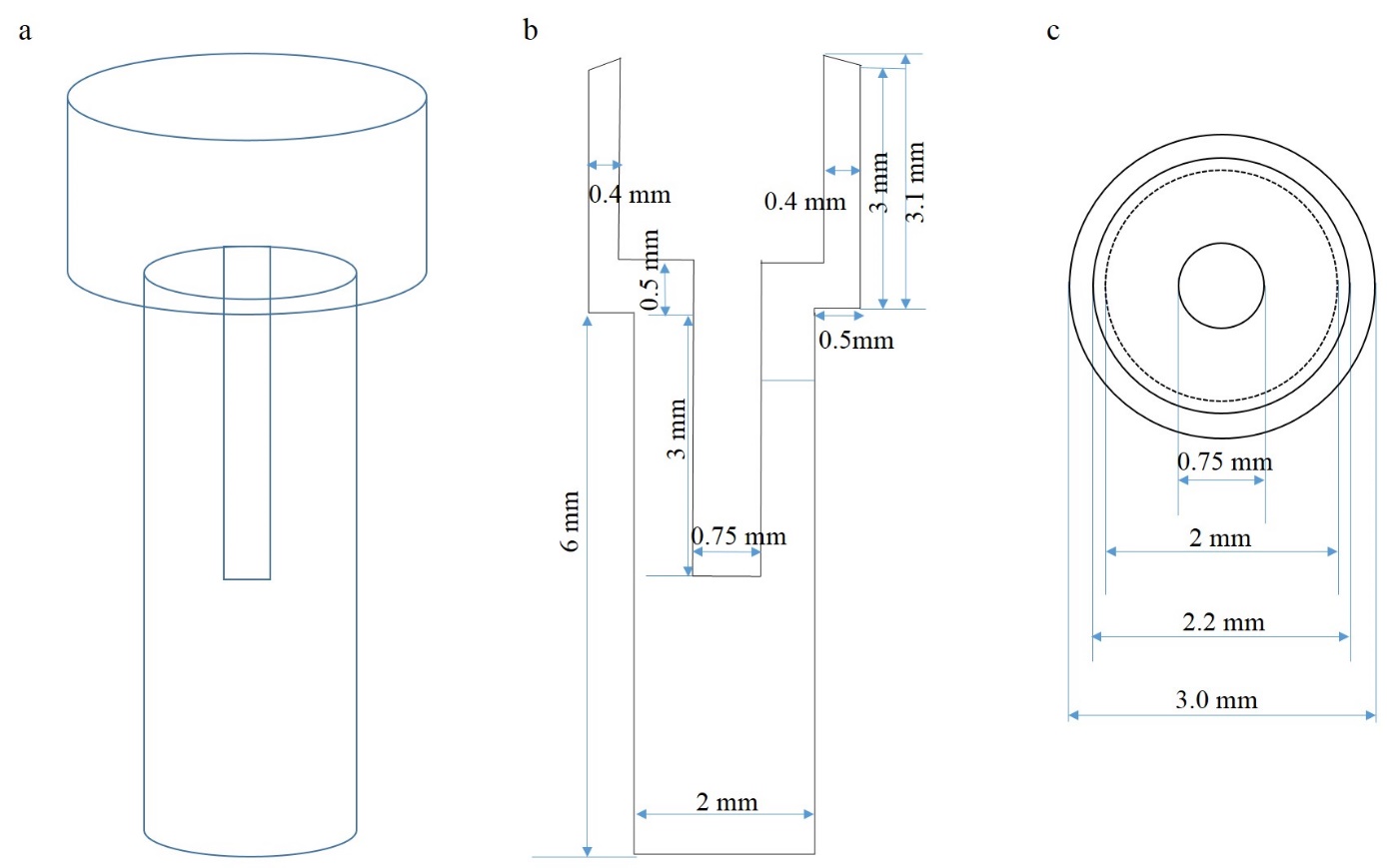


**Figure S4. In-house design of the sample holder**. **a** Scheme diagram of the holder. **b** Front view. **c** Top view.


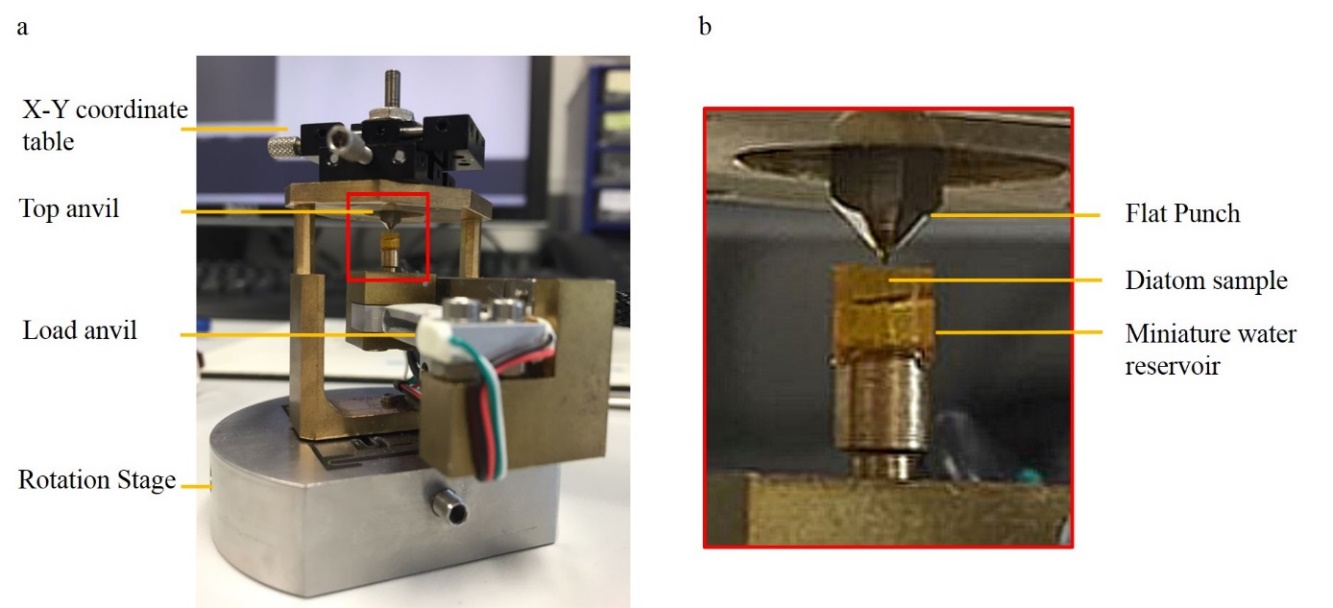


**Figure S5. In-situ compression test on diatoms in wet state. a** Sample stage with compression loading option. **b** Magnified image of sample area (red rectangle in **a**).


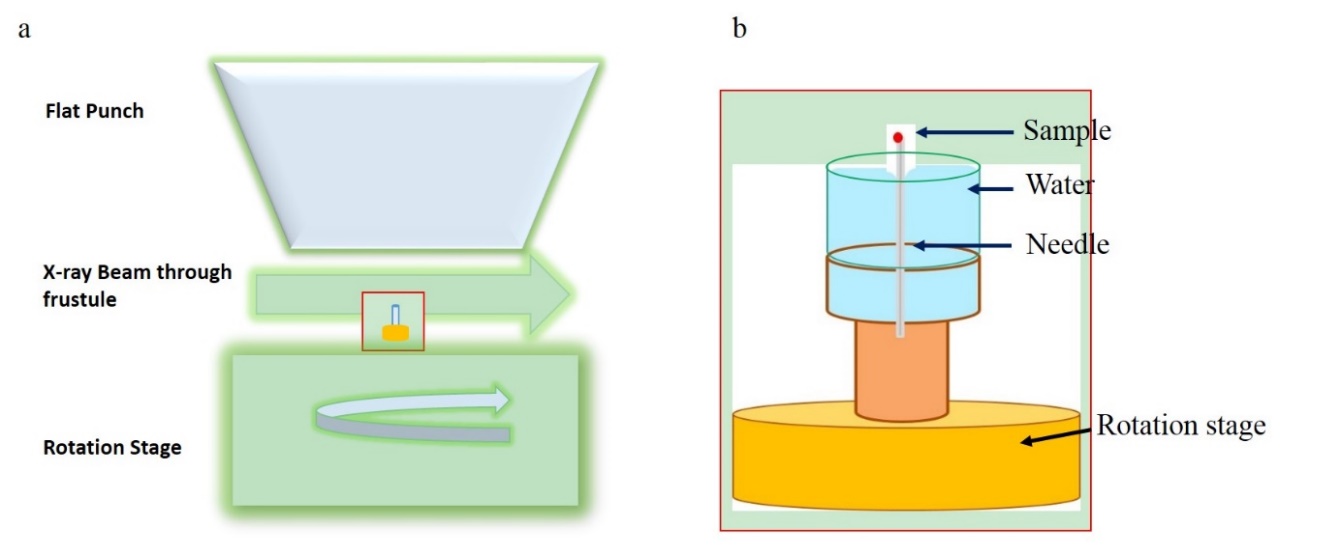


**Figure S6. Set-up of the in-situ compression test of the diatom cell in wet state**. **a** Scheme of the micro-mechanical test set-up integrated into a nano-XCT tool. **b** Scheme of the sample part in the in-situ compression test set-up.


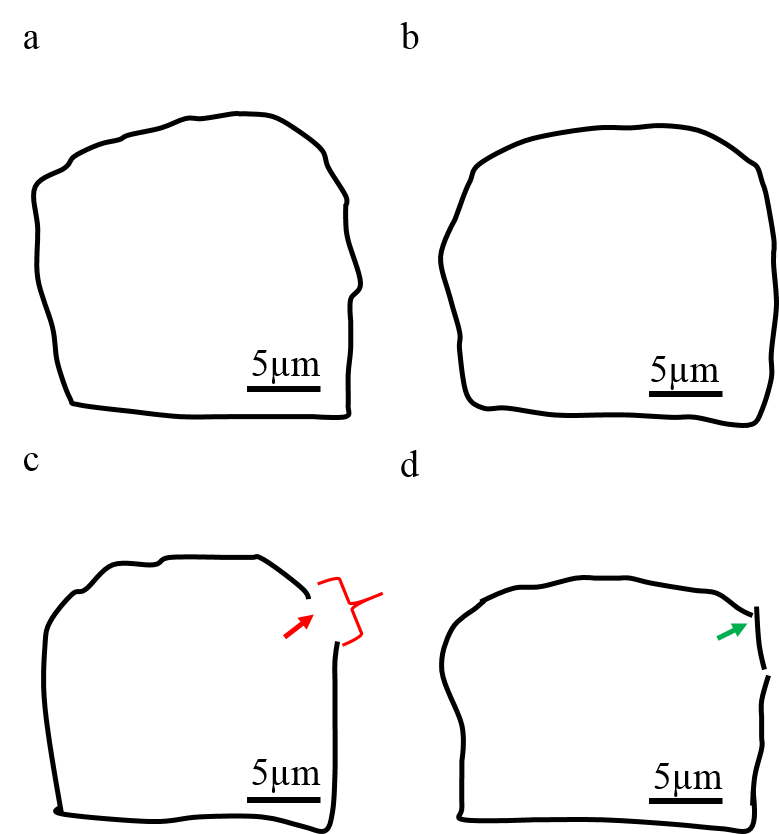


**Figure S7. The indicated sketch of the studied diatom in Fig. 4 for the in-situ compression test on *Actinocyclus* sp. in dry state.** **a** Outline map of the first contact between the flat punch and the cell. **b** Outline map before the micro-crack appeared. **c** Outline map of the micro-crack position. **d** Outline map of the cell with further compression after the micro-crack appeared. Red arrows: the micro-crack within the compression test. Red right brace: the crack opening and size. Green arrow: the cell is further deformed by the compression.

**
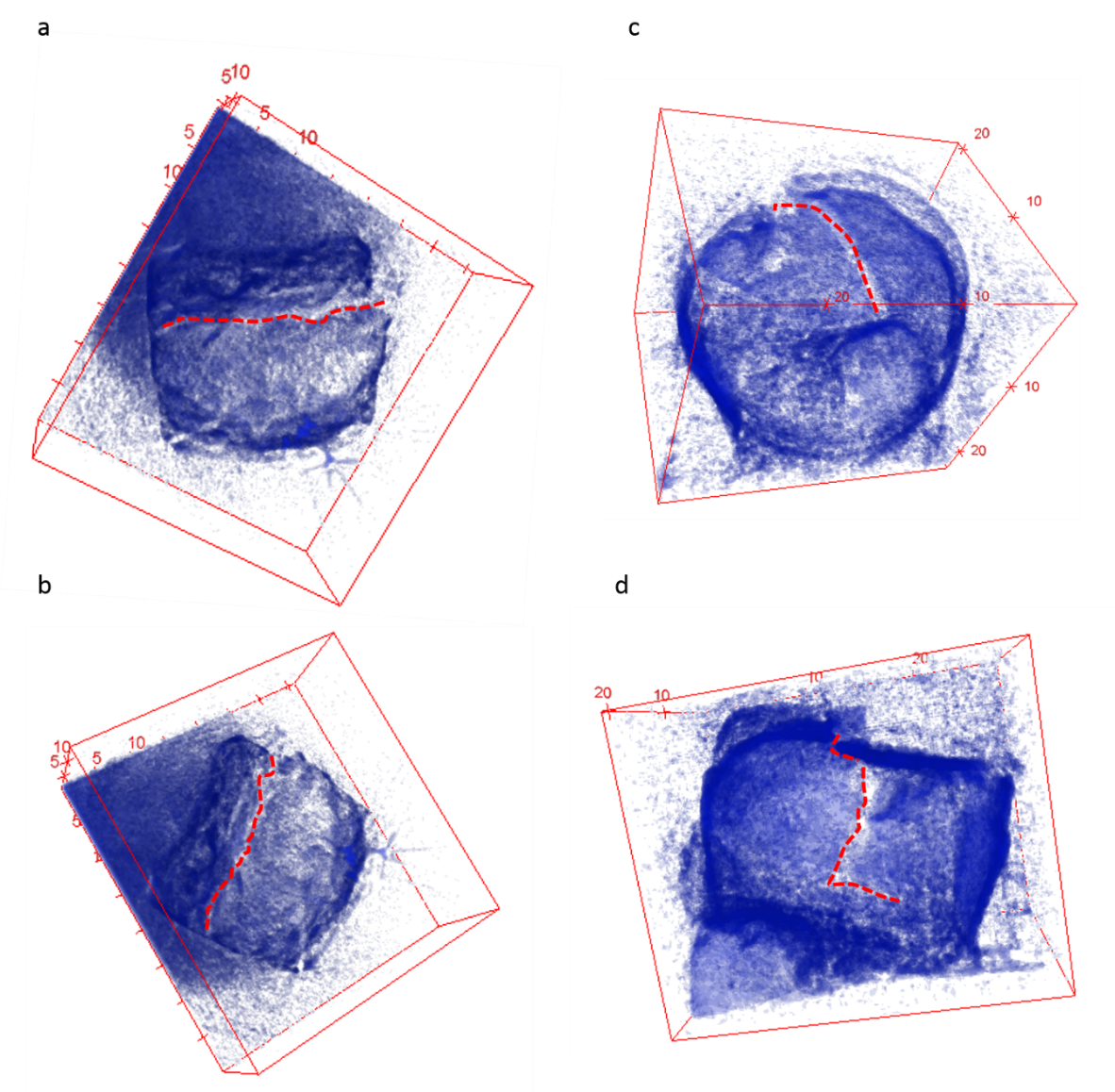
**

**Figure S8. 3D volume rendering images of the whole diatom frustule after in-situ compression test in the nano-XCT tool in both states.** **a,** **b** The 3D rendering imaging of *Actinocyclus* sp after the compression test in wet state. **c,** **b** The 3D rendering imaging of *Actinocyclus* sp after the compression test in dry state. Red dashed lines: the positions of the cracks and their propagations.

**Table S1. Nutrient solution for diatoms: 1L recipe.**

| Component | Amount to add |
| --- | --- |
| Medium concentrated f/2 ^1^ solution | 1 ml |
| Concentrate silicate solution | 1 ml |
| Coral salt* | 35 g |
| Solution final | 1 L |
| Solution of each Erlenmeyer flasks | 125 ml |

* Coral salt is Coral reef and got from https: //aquaforest.eu/de/product/reef-salt.
